# Pro-inflammatory cytokines play a key role in the development of radiotherapy-induced gastrointestinal mucositis

**DOI:** 10.1186/1748-717X-5-22

**Published:** 2010-03-16

**Authors:** Zhi Yi Ong, Rachel J Gibson, Joanne M Bowen, Andrea M Stringer, Jocelyn M Darby, Richard M Logan, Ann SJ Yeoh, Dorothy M Keefe

**Affiliations:** 1 School of Medicine, University of Adelaide, Adelaide, South Australia; 2 School of Medical Sciences, University of Adelaide, Adelaide, South Australia; 3 School of Dentistry, University of Adelaide, Adelaide, South Australia; 4 Cancer Council South Australia, 202 Greenhill Road, Eastwood, South Australia

## Abstract

**Background:**

Mucositis is a toxic side effect of anti-cancer treatments and is a major focus in cancer research. Pro-inflammatory cytokines have previously been implicated in the pathophysiology of chemotherapy-induced gastrointestinal mucositis. However, whether they play a key role in the development of radiotherapy-induced gastrointestinal mucositis is still unknown. Therefore, the aim of the present study was to characterise the expression of pro-inflammatory cytokines in the gastrointestinal tract using a rat model of fractionated radiotherapy-induced toxicity.

**Methods:**

Thirty six female Dark Agouti rats were randomly assigned into groups and received 2.5 Gys abdominal radiotherapy three times a week over six weeks. Real time PCR was conducted to determine the relative change in mRNA expression of pro-inflammatory cytokines IL-1β, IL-6 and TNF in the jejunum and colon. Protein expression of IL-1β, IL-6 and TNF in the intestinal epithelium was investigated using qualitative immunohistochemistry.

**Results:**

Radiotherapy-induced sub-acute damage was associated with significantly upregulated IL-1β, IL-6 and TNF mRNA levels in the jejunum and colon. The majority of pro-inflammatory cytokine protein expression in the jejunum and colon exhibited minimal change following fractionated radiotherapy.

**Conclusions:**

Pro-inflammatory cytokines play a key role in radiotherapy-induced gastrointestinal mucositis in the sub-acute onset setting.

## Introduction

Mucositis is a debilitating side effect of cytotoxic chemotherapy (CT) and radiotherapy (RT). It involves inflammation and mucosal ulceration of the alimentary tract, resulting in symptoms including pain, abdominal bloating, nausea, vomiting and diarrhoea [1-3]. The effects of mucositis often limit the dose of cytotoxic agents that can be administered and in some cases, even prevents patients from undergoing further treatment to control the malignancy [4].

It has been postulated that mucositis occurs in five overlapping phases: initiation, upregulation and message generation, signalling and amplification, ulceration and healing [5]. Nuclear factor kappa B (NFκB), cyclooxygenase-2 (COX-2) as well as pro-inflammatory cytokines (in particular interleukin (IL)-1β (IL-6) and tumour necrosis factor (TNF)) have been suggested to play a key role in this 5 phase mucositis model [5].

Previous research has clearly shown that IL-1β, IL-6 and TNF are upregulated in the buccal mucosa, jejunum and colon of rats following administration of chemotherapy [6]. Furthermore, elevated levels of IL-1β and TNF have been detected in the buccal mucosa of hamsters who received combined chemotherapy and radiotherapy [7,8]. In addition, various studies have attempted to target pro-inflammatory cytokines as a preventive measure for intestinal mucositis [8-11]. For example, palifermin and IL-11 have been reported to be successful in lowering the levels of pro-inflammatory cytokines in the development of mucositis [8-11]. Furthermore, they also attenuate mucositis in animal models [8-12], thus supporting the current view that pro-inflammatory cytokines play a major role in the development of mucositis.

Recently, we have developed a fractionated radiotherapy-induced mucositis model in the Dark Agouti (DA) rat [13]. The model involves rats receiving one to six weeks of radiotherapy. In the clinical setting, fractionated radiotherapy is usually more common than a single high dose. Thus, this model provides the ideal opportunity to explore various avenues involved in fractionated radiotherapy-induced mucositis, with rats receiving between one and three weeks of radiotherapy representing short-term, and those receiving between four and six weeks representing long-term radiotherapy in the clinical setting [13]. Damage which occurs in the short term is an acute event, while damage in the long term is considered sub-acute. Histological damage peaks mid treatment and begins to subside towards the completion of radiotherapy, despite worsening clinical symptoms of intestinal toxicity [14]. The cause of this is unknown but may be related to inflammatory changes. Therefore the aim of the present study was to characterise the expression of pro-inflammatory cytokines in the intestines during six weeks of fractionated radiotherapy. We hypothesise that pro-inflammatory cytokine levels in the jejunum and colon will be elevated following radiotherapy and that this increase will correlate with the increasing duration and total doses of radiotherapy.

## Methods

### Ethics

This study was approved by the Animal Ethics Committee of the Institute of Medical and Veterinary Sciences, Adelaide and the University of Adelaide. Animal work and handling were complied with the National Health and Research Council (Australia) Code of Practice for Animal Care in Research and Teaching (2004) [13].

### Irradiation Protocol and Experimental Design

Thirty six female DA rats (150 g - 170 g) were obtained from the University of Adelaide Breeding Facility. All animals were maintained in an environmentally controlled condition of 12-h light/12-h dark cycles and allowed free access to food and water. Rats were randomly assigned to groups based on RT dose as follows: Control (no treatment); 7.5 Gy; 15 Gy; 22.5 Gy; 30 Gy; 37.5 Gy and 45 Gy (Table 1). Detailed radiation procedures have been described previously [13]. Briefly, rats were anaesthetised prior to receiving 2.5 Gys of radiotherapy to the abdomen three times a week for up to six weeks.

**Table 1:** Experimental Design.

Group	Rat Number	Treatment Duration (Weeks)	Total Radiation Dose (Gy)
1	n = 5	1	7.5
2	n = 5	2	15
3	n = 5	3	22.5
4	n = 5	4	30
5	n = 5	5	37.5
6	n = 5	6	45
Control	n = 6	6	0

Groups of rats (n = 5) were exposed to varying doses of fractionated radiotherapy over a six week period. Control rats (n = 6) received no fractionated radiotherapy.

### Tissue Collection

Rats were killed by exsanguination followed by cervical dislocation and the entire gastrointestinal tract removed. The small and large intestines were separated and flushed with chilled saline to remove intestinal contents. Sections of jejunum (collected at 33% of the length from the pyloric sphincter) and colon (collected at 50% of the length) were collected and either fixed in 10% neutral buffered formalin and embedded in paraffin for histopathology and immunohistochemistry or snap frozen with liquid nitrogen and stored at -70°C for real time PCR.

### Histopathology

Routine histopathological examination using standard haematoloxylin and eosin staining was conducted. These methods have previously been validated and described elsewhere [15].

### RNA extractions

Total RNA was isolated and purified using the NucleoSpin^® ^RNA II kit (Macherey-Nagel, Duren, Germany) following manufacturer's instructions. The integrity of RNA extracted was determined by comparing sharp 28S and 18S rRNA bands electrophoresed on a 1.5% formaldehyde gel and 260/280 ratios.

### Reverse Transcription

1 μg RNA was reverse transcribed to generate cDNA using the iScript™ cDNA Synthesis Kit (Bio-Rad Laboratories, Hercules, CA) according to manufacturer's instructions. 100 ng of cDNA from each sample was subsequently used in real time PCR.

### Real Time PCR

The amplification reactions were conducted in a volume of 10 μL containing 1× Quantitect SYBR Green master mix (Qiagen) forward and reverse primers each at a final concentration of 2.5 ng/μL and 100 ng cDNA. Primer sequences for IL-1β, IL-6, TNF and β-actin are stated in Table 2. Real time PCR was carried out using Rotor-Gene 6000 real time rotary analyser (Corbett Life Science, Sydney, Australia). Taq DNA polymerase was activated at 95°C for 10 minutes followed by 45 cycles of denaturing at 95°C (15 s) and annealing/extension at 60°C (1 min). Relative quantification of mRNA expression was performed using the Delta Delta C_t _(2^-ΔΔCT^) method (also known as the comparative C_t _method) as described in Livak and Schmittgen (2001) [16], using the Rotor-Gene software. To improve sample size the original groups were pooled into larger short term (3 weeks or less RT) and long term (4 weeks or greater RT) groups.

**Table 2:** Primer sequences for IL-1β, IL-6, TNF and β actin.

Gene	Primer Sequence	Size (bp)	Accession No
**IL-1β**	Forward: 5'-CACCTCTCAAGCAGAGCACAGA-3'	81	NM_031512
	Reverse: 5'-ACGGGTTCCATGGTGAAGTC-3'		

**IL-6**	Forward: 5'-ATATGTTCTCAGGGAGATCTTGGAA-3'	80	NM_031512
	Reverse: 5'-GTGCATCATCGCTGTTCATACA		

**TNF**	Forward: 5'-GTGATCGGTCCCAACAAG-3'	71	X66539
	Reverse: 5'-AGGGTCTGGGCCATGGAA-3'		

**β actin**	Forward: 5'-AGGCCAACCGTGAAAAGATG-3'	101	NM_031144
	Reverse: 5'-ACCAGAGGCATACAGGGACAA-3'		

### Immunohistochemistry

Four micron tissue sections were dewaxed with xylene and rehydrated through decreasing concentrations of alcohol. Endogenous peroxidase was blocked with 0.5% hydrogen peroxide in methanol for 20 minutes. This was followed by antigen retrieval in citrate buffer (pH 6.0) heated in a microwave at high power (900W) (3 min) and low power (650W) (10 min). Non-specific binding was blocked with 50% normal goat or horse serum in PBS (pH 7.5) (Sigma-Aldrich Inc, St. Louis, MO) Avidin and biotin was blocked using the avidin and biotin blocking solution (Vector Laboratories, Burlingame, CA). Primary antibodies (IL-1β: Rabbit Polyclonal, Santa Cruz Laboratories, 1.100 dilution; IL-6: Rabbit Polyclonal, Santa Cruz Laboratories, 1:1000 dilution; TNF: Goat Polyclonal antibody, Hycult Biotechnology, 1:250 dilution) were applied to sections and incubated at 4°C overnight. Primary antibody incubations were omitted for negative controls. Sections were incubated in biotinylated secondary antibody followed by ultrastreptavidin peroxidase (Signet Pathology Systems Inc., Dedham, MA). Antibodies were visualized with diaminobenzidine (DAB) (Zymed laboratories, San Francisco, CA). Sections were counterstained with Lillie Mayer's haematoxylin, dehydrated, cleared in xylene, coverslipped and viewed using light microscopy. Staining intensity was graded according to a previously published and validated grading system where 0 = no staining, 1 = weak staining, 2 = moderate staining, 3 = strong staining, 4 = very intense staining [6,13,17].

### Statistical Analysis

Statistical analyses were conducted using either one-way ANOVA followed by Tukey's Post Hoc test, or Kruskal Wallis test followed by Dunn's Post Hoc test. Results were deemed significant should p < 0.05.

## Results

### Histopathology

Pathological changes over time in the rat intestinal tract caused by fractionated radiotherapy have previously been described in detail [13]. Briefly there was no histopathological change at any time point in rats that did not receive radiotherapy. However, rats that received radiotherapy had an increase in apoptosis in the jejunum and colon, as well as severe goblet cell disintegration. Furthermore, there was a significant alteration in the height of the jejuna and colonic crypts over the radiotherapy course [13].

### Change in mRNA expression of pro-inflammatory cytokines in the jejunum

The mRNA expression of IL-1β, and TNF in rats receiving fractionated radiotherapy did not differ significantly from the expression in control animals. However, IL-6 mRNA levels were increased (although did not reach significance) in rats receiving 45 Gy of RT compared with all other doses (data not shown).

**Figure 1:**
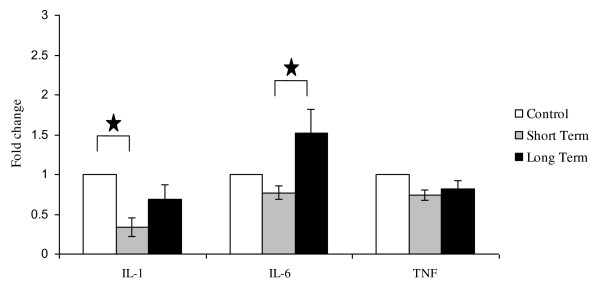
**mRNA expression of IL-1β, IL-6 and TNF in the Jejunum of DA rats in the following groups: untreated controls, short term course of radiotherapy (Weeks 1 - 3), long term course of radiotherapy (Weeks 4 - 6)**. Data are expressed as mean + SEM. There was a significant decrease in IL-1 expression between short-term radiotherapy groups and controls (p < 0.05). There was a significant increase in IL-6 between long-term radiotherapy and short-term radiotherapy groups (p < 0.05).

When the data was pooled, mRNA expression of IL-1β was significantly less in the short term RT group compared with controls (Figure 1). IL-6 mRNA levels in the long term RT group were significantly higher than the short term RT group (Figure 1). No significant differences in TNF mRNA levels were observed.

### Change in mRNA expression of pro-inflammatory cytokines in the colon

The mRNA expression of IL-1β, IL-6 and TNF in rats receiving fractionated radiotherapy did not differ significantly from the expression in control animals. However, IL-1β levels in rats receiving 37.5 Gy and 45 Gy RT were increased (although did not reach significance) compared to all other groups (data not shown).

**Figure 2:**
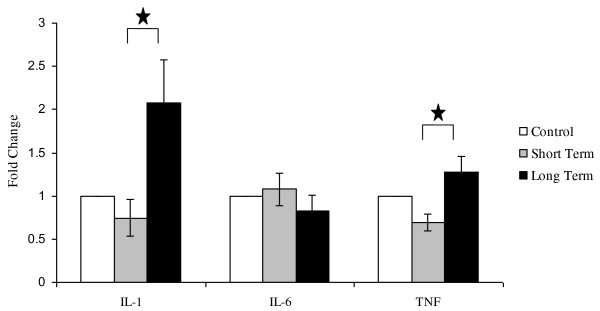
**mRNA expression of IL-1β, IL-6 and TNF in the Colon of DA rats in the following groups: untreated controls, short term course of radiotherapy (Weeks 1 - 3), long term course of radiotherapy (Weeks 4 - 6)**. Data are expressed as mean + SEM. There was a significant increase in both IL-1β and TNF expression between short term and long term radiotherapy groups (p < 0.05).

When these individual groups were grouped together, there was significantly greater IL-1β mRNA expression in rats receiving long term radiotherapy than in rats receiving short term radiotherapy (Figure 2). IL-6 mRNA levels of rats in the control, short term and long term radiotherapy groups did not differ significantly (Figure 2). Rats receiving long term radiotherapy demonstrated a significantly higher TNF mRNA expression from rats which received short term radiotherapy (Figure 2).

### Expression of pro-inflammatory cytokines in the jejunum and colon

#### IL-1β

In general, there was weak-moderate IL-1β staining in the jejunal crypts. There was predominantly weak staining of the villi. The intensity of IL-1β staining fluctuated throughout six weeks of radiotherapy (Data not shown). IL-1β staining intensity in the colon was weak-moderate over six weeks of radiotherapy. Staining was variable between the basal and apical regions of the crypts and did not significantly change of the course of radiotherapy (Data not shown).

#### IL-6

IL-6 staining was weak-moderate in the crypts of the jejunum and weak in the villi. No differences in IL-6 expression were observed over six weeks of radiotherapy (Data not shown). IL-6 expression in the colon did not change over six weeks of radiotherapy. IL-6 staining intensity in the basal region of the crypt (moderate to strong) was slightly higher than the apical region (weak to moderate) (data not shown).

#### TNF

**Figure 3:**
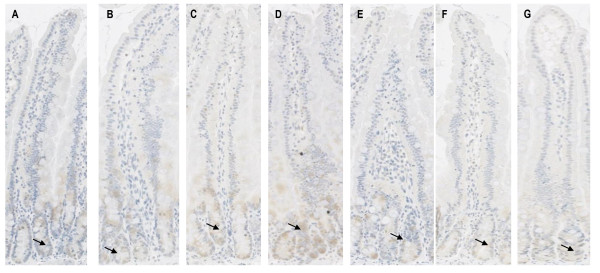
**Protein expression of TNF in the jejunum following six weeks of fractionated radiotherapy**. A = control; B = 7.5 Gy; C = 15 Gy; D = 22.5 Gy; E = 30 Gy; F = 37.5 Gy G = 45 Gy. There was no change in the level of expression at any time point. Staining was only observed in the crypts, as indicated by the arrows. There was no staining seen in the villi.

**Figure 4:**
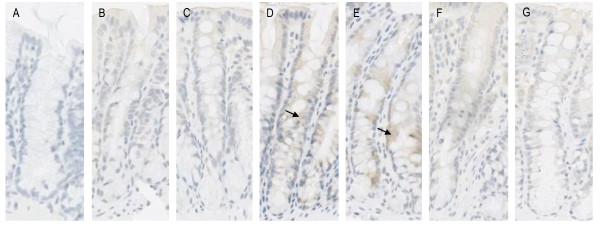
**Protein expression of TNF in the colon following six weeks of fractionated radiotherapy**. A = control; B = 7.5 Gy; C = 15 Gy; D = 22.5 Gy; E = 30 Gy; F = 37.5 Gy G = 45 Gy. No staining was seen in the crypts of rats that had received no radiotherapy. There was an increase in protein expression of TNF after radiotherapy, particularly after 22.5 Gy and 30 Gy as indicated by the arrow, although the staining was not considered to be very strong.

TNF staining was moderate in the jejuna crypts. No staining was seen along the villi. TNF protein levels did not appear to differ among individual groups of rats which underwent one to six weeks of radiotherapy and controls (Figure 3). No TNF was expressed in the colon of rats that had not received radiotherapy. TNF expression increased slightly over the course of six weeks of radiotherapy, being particularly evident after 22.5 Gy and 30 Gy. (Figure 4). There was more TNF staining observed towards the basal region of the crypt.

#### Submucosal protein expression of IL-1β, IL-6 and TNF

All tissue sections were assessed for the submucosal protein expression of IL-1β, IL-6 and TNF. There was no apparent submucosal staining in the vast majority of sections. Occasional sections had positive staining in blood vessels and in the cells of the lamina propria (data not shown).

## Discussion

This study has shown for the first time, using the fractionated radiotherapy-induced mucositis rat model, that mRNA levels of pro-inflammatory cytokines, IL-1β, IL-6 and TNF, are significantly upregulated in the intestines following long term radiotherapy when compared to short term radiotherapy. Significant reductions in IL-1β mRNA levels were found in the jejunum during short term radiotherapy. The upregulation of pro-inflammatory cytokine mRNA levels was seen in rats receiving either five or six weeks of radiotherapy, and supports the Sonis [5] hypothesis that pro-inflammatory cytokines increase with increasing fractionated radiotherapy. Furthermore, the elevated levels of pro-inflammatory cytokines following five and six weeks of radiotherapy correlates with histological evidence of intestinal mucositis and peak expression of NFκB [13]. Together, these findings strongly suggest that long term radiotherapy is capable of activating NFκB, which subsequently stimulates increased production of pro-inflammatory cytokines in the intestines leading to greater tissue damage. This study also demonstrated decreased pro-inflammatory cytokine levels in the intestines of rats receiving one to three weeks, or short term radiotherapy. Rats undergoing short term radiotherapy showed a significant reduction in IL-1β mRNA levels and, to a lesser extent, IL-6 and TNF, when compared to rats receiving no radiotherapy. These observations are in contrast with previous findings where pro-inflammatory cytokine mRNA levels in the gastrointestinal tract were found to be elevated five days following chemotherapy in rats and 12 days post-radiation treatment in hamsters [7,8]. The changes in pro-inflammatory cytokine levels encountered in this current study may be due to the differential effects of short term and long term courses of radiotherapy, in which long term radiotherapy exerts pro-inflammatory effects as observed in high dose radiation while short term radiotherapy may mimic the anti-inflammatory effects seen in low dose radiation [18]. Radiation exposure in the range 1-2 Gy is known to activate the growth stimulatory ERK pathway via EGFR [19]. It has been suggested that this activation is mediated through radiation-induced free radicals [19]. Free radicals are also strongly linked to activation of NFκB and the pro-inflammatory pathway, as well as JNK signalling [20], indicating a balance between outcomes which is highly dose-dependent and linked to free-radical generation.

The paradoxical findings of this study may be best explained by the degree of damage present in the short and long term radiotherapy setting. Low dose radiation is known to induce apoptosis, a process that suppresses inflammation via signals released by engulfing phagocytes. However, in areas of intense damage there is often increased necrosis, whereby cells release factors serving as potent stimuli for inflammation. Increasing duration of radiation may have led to a depletion of cytosolic pools of NAD and ATP in the intestinal cells, resulting in a switch from apoptosis to necrosis [20] at the later time points, consequently activating pro-inflammatory cytokines as reflected in our results.

When pro-inflammatory cytokine levels were examined at the protein level, we saw no significant changes in the intestinal epithelium of rats receiving radiotherapy. Previous research into expression at the protein level has shown conflicting findings. In one study, pro-inflammatory cytokine protein levels in the epithelium throughout the gastrointestinal tract were upregulated as early as six hours after chemotherapy [6]. However, another study demonstrated an increase in the protein expression of IL-1β in the oral submucosa and not in the epithelium following radiotherapy [8]. These discrepancies may be the result of different treatments. Chemotherapy is given systemically and is generally only administered for a single short period with the resulting, mucosal injury usually acute [5]. Radiotherapy, on the other hand, is a localised treatment and can cause both acute and chronic injury [5,21]. The present study utilised fractionated radiotherapy. This model is more clinically relevant compared to other single dose radiotherapy models as fractionated radiotherapy is more commonly given to cancer patients. Fractionated radiotherapy not only kills tumour cells more effectively, it also allows normal cells to repair and regenerate in between fractions, making them more tolerant to radiation and less prone to radiation-induced damage [21]. Our previous studies using fractionated radiotherapy showed an increase in crypt length following two to six weeks of radiotherapy [13]. This observation is exclusive to radiotherapy as our previous studies utilising chemotherapy have reported a reduction in crypt length [22,23]. Therefore it is likely in this study that the crypt cells initiated compensatory mechanisms enabling them to repair and repopulate, resulting in increased crypt length as well as unchanged pro-inflammatory cytokines protein levels seen in the intestinal epithelium.

In conclusion, this novel fractionated radiotherapy-induced mucositis model has allowed the characterisation of pro-inflammatory cytokines IL-1β, IL-6 and TNF in the jejunum and colon of the DA rat following radiotherapy, thus confirming the importance of these cytokines in the development of mucositis. Pro-inflammatory cytokines were upregulated at later time points of radiotherapy suggesting that these cytokines can ultimately induce more tissue injury and inflammation in the intestine with increasing total doses of radiotherapy. Expression was altered in the epithelial compartment (not sub-epithelial regions) indicating enterocyte upregulation rather than infiltrating immune cells. As such, the pathophysiology of fractionated radiotherapy-induced mucositis is different to immune-regulated inflammatory bowel disease. However, more research is still required to clarify the localisation of these cytokines and the molecular mechanisms involved in the development of mucositis.

## Competing interests

The authors declare that they have no competing interests.

## Authors' contributions

ZYO carried out the real-time PCR and immunohistochemistry staining and assisted in manuscript preparation. RJG participated in the study design, assisted in the animal studies, performed data analysis and was responsible for the overall manuscript preparation. JMB participated in the study design, assisted in the animal studies, assisted in the conduction of the real-time PCR and assisted in manuscript preparation. AMS participated in the study design, assisted in the animal studies, performed data analysis and assisted in manuscript preparation. JMD was responsible for slide analysis and image presentation. RML participated in the study design and assisted in the animal studies. ASJY participated in the study design, conducted the animal studies, and carried out the histopathology. DMK conceived of the study and participated in its design and coordination. All authors read and approved the final manuscript.
